# Modelling Key Performance Indicators (KPIs) in the Optimisation of Nanoimprint Lithography (NIL) Processes

**DOI:** 10.3390/mi17040491

**Published:** 2026-04-17

**Authors:** Andrzej Pacana, Karolina Czerwińska

**Affiliations:** Faculty of Mechanical Engineering and Aeronautics, Rzeszow University of Technology, Al. Powstancow Warszawy 12, 35-959 Rzeszow, Poland; k.czerwinska@prz.edu.pl

**Keywords:** nanofabrication, thin films, throughput enhancement, manufacturing performance metrics, indicator analysis, mechanical engineering, data-driven manufacturing

## Abstract

Nanoimprint lithography (NIL) plays an increasingly important role in modern nanomanufacturing processes, but its effective application in production conditions requires precise tools for evaluating and optimising technological processes. The aim of the study was to develop and model key performance indicators (KPIs) supporting the optimisation of the quality, stability and efficiency of nanoimprint lithography processes. As part of the selection of indicators, a literature review, surveys and in-depth interviews with industry experts were conducted, which enabled the identification of indicators relevant from a technological practice perspective. The proposed KPI classification was directly linked to the stages of the NIL process, creating a basis for operational performance control and process capability analysis. A novel aspect is the proposal of an integrated KPI model that combines the classification of indicators based on the stages of the NIL process with their direct link to technological parameters and measurable quality effects. These indicators have been linked to critical process parameters for different NIL variants, including Thermal NIL, UV-NIL, Roll-to-Roll NIL and Step-and-Repeat NIL, using a process analysis and multi-criteria optimisation approach. Research indicates that the use of an integrated KPI model improves the accuracy of nanostructure mapping, reduced defect density, and increased process efficiency without compromising technological stability. The proposed approach is a universal tool supporting NIL process control, its scaling to industrial applications, and integration with statistical process control and data-driven optimisation methods.

## 1. Introduction

Dynamic progress in microelectronics, particularly in relation to the growing demands for the miniaturisation of integrated circuits (ICs) and optoelectronic devices, has made micro- and nano-manufacturing technologies an important factor determining the development of modern technological solutions [1,2,3,4]. In this context, photolithography is one of the most important stages in the semiconductor manufacturing process. For many years, it has been subject to further refinement and systematic optimisation [5,6,7,8]. Its conventional variants, including deep ultraviolet (DUV) lithography, form the foundation of modern integrated circuit production. This is due to their widespread use in the formation of microstructures with high precision and resolution [9,10,11]. Extreme ultraviolet (EUV) lithography, developed in parallel, enables nanometre-scale technology to be implemented. However, this technology is associated with high technological infrastructure costs and significant manufacturing complexity [12,13,14,15,16,17]. As a result, nanoimprint lithography (NIL) is considered a complementary solution that can supplement conventional lithographic methods. NIL is a promising technology for producing nanoscale patterns within thin protective layers applied to substrates. This process is based on compression moulding and has great potential for mass production [18].

Nanoimprint lithography, proposed by Chou in 1995, is considered a promising technology with significant potential for application [19,20]. NIL can be viewed as a micro-injection process in which the printed features are defined by the template topography and involve the physical deformation of a thin resist layer using a hard mould at high temperature and pressure [21]. NIL technology does not involve chemical modification of the photoresist under the influence of radiation; therefore, the resolution of the reproduced structures is much less susceptible to factors limiting the performance of conventional lithography. These factors include: scattering and interference in the photoresist, wave diffraction, backscattering from the substrate, and the chemical properties of the photoresist and developer themselves [22].

NIL technology, as a method of pattern transfer, has gained considerable attention in recent years in both academic and industrial circles [23,24,25,26,27,28]. Compared to conventional lithographic techniques, NIL enables high-resolution patterns to be obtained at scales below 100 nanometres, and in some cases even smaller, while avoiding the limitations of diffraction or standing-wave effects in the lithographic process [29,30]. In addition, NIL can be used with relatively inexpensive equipment and materials, making it an attractive alternative for industrial applications. This method is also characterised by high reproduction precision, process simplicity and high production efficiency [31].

A thorough understanding of the basic physical and chemical mechanisms involved in NIL processes is important for the further development of this technology. Computational simulations and theoretical studies are effective tools for obtaining new results in situations that are difficult to investigate experimentally. The literature on the subject indicates increased research activity in the field of improving various variants of NIL technology. Shin et al. conducted both experiments and numerical simulations to deepen their knowledge of the behaviour of polymer materials and the effective optimisation of the Thermal NIL (T-NIL) process. The analysis accounted for the influence of process parameters on polymer deformation. The results made it possible to identify the conditions enabling a fast, stable and efficient thermal printing process [32].

Simulation models based on continuum mechanics are being developed to analyse polymer deformation under process conditions. The results of the simulation studies, supported by experimental data, have confirmed that it is possible to produce patterns measuring 100 nanometres × 860 nanometres using thick polymer layers [33]. Molecular dynamics (MD) simulations were also performed to analyse the relationship between the pressure required to fill the cavity of a polymethyl methacrylate (PMMA) mould in the T-NIL process [34,35]. The results of the study showed a significant influence of the size (e.g., chain length) of the polymer on the NIL process, which was not taken into account in models based on continuum mechanics.

Extensive simulation studies of ultraviolet NIL (UV-NIL) processes were conducted, including photoresist filling and evaluation of the mechanical properties and shrinkage of UV-cured photoresists, using a conventional continuum mechanics approach [36]. Numerical modelling studies using the finite element method were also carried out, which showed that the effectiveness of the UV-NIL process depends to a large extent on the selection of the optimal concentration of the cross-linking agent [37]. These simulations, based on a continuum approach, provided important information on NIL processes with a resolution of 100 nm. Currently, the resolution of NIL patterns is below 10 nm. Therefore, analysing the NIL process at the atomic scale is essential for further development in this field. At this scale, the mechanical properties of structures are strongly determined by molecular characteristics and the behaviour of resists.

In one study [38], atomistic molecular dynamics (MD) simulations of the cavity filling process in UV-NIL technology were performed to assess the impact of resist properties on the reproduction of patterns with a resolution below 10 nanometres. The filling of silicon grooves with a width of 1–3 nanometres by resist materials of varying viscosity under constant pressure was analysed. The results showed that lower viscosity promotes effective and faster cavity filling, and in multicomponent systems, molecules with lower viscosity preferentially penetrated the narrowest grooves, leading to compositional heterogeneity and potential defects after UV curing. It was also found that the presence of small molecules facilitates the penetration of multifunctional molecules into narrow structures, which is beneficial for obtaining high-resolution patterns. The results provide important guidance for designing resists with limited susceptibility to defects.

The roll-to-plate (R2P) method in nanoimprint lithography is a hybrid solution between continuous and discrete processes, in which the nanostructure pattern is transferred from a flexible cylinder onto a rigid, flat substrate (such as silicon wafers or glass). This process can be carried out using both Thermal and UV methods. The advantages of R2P include the ability to process larger areas than with Step-and-Repeat methods and relatively good structural reproduction quality [39]. However, the limitations of this method—related to mechanical complexity, ensuring uniform pressure, and the lack of full process continuity—mean that R2P is less efficient than the NIL techniques more widely used in industry. As a result, roll-to-plate is used in selected, specialised fields, such as optics or surface functionalisation, and remains much less widely used in industry than dominant variants, such as Roll-to-Roll NIL or UV-NIL, due to its niche nature [40].

Advances in the optimisation of NIL processes, as well as the development of its various variants, translate into an expansion of the spectrum of applications for this technology in areas requiring high-resolution and precision mapping. In recent years, NIL technology has found wide application in the production of photonic and electronic components, including photonic crystals, lenses and optical gratings, in which the integration of nanostructures leads to improved functional parameters [41]. NIL technology has become particularly important in the field of patterned sapphire substrates (PSSs) [42], LED structures [43] and photovoltaic cells [44], where its technological advantages are clearly visible.

In the production of patterned sapphire substrates (PSSs), NIL technology enables large-scale manufacturing processes while reducing costs, eliminating the need for expensive lithography equipment and complex masking processes [45,46]. A sapphire substrate with a precisely shaped microstructure is crucial in the manufacture of LED integrated circuits, and control of the surface microstructure is critical to increasing the optoelectronic efficiency of LEDs [47,48,49]. Similar benefits are found in the production of LED integrated circuits and photovoltaic cells, where low costs, high efficiency and precise pattern transfer make NIL technology a valuable addition to industrial processes [50,51]. This is particularly important in applications where extreme resolution requirements are less stringent than in traditional lithographic techniques. In such situations, NIL technology reduces manufacturing costs, simplifies complex production steps, and increases process efficiency while maintaining high pattern reproduction accuracy [52].

The prospects for the application of nanoimprint lithography technology and its variants are very broad. NIL technology is used in many fields that require high precision and nanoscale control, where the integration of nanostructures improves the functionality and performance of devices [53,54]. However, the effective use of NIL’s potential and the achievement of the expected benefits of its implementation require systematic monitoring and optimisation [55]. Key performance indicators (KPIs) are an important tool in this context. Properly designed indicators enable the assessment of mapping quality and ensure process stability and production efficiency. The implementation of KPIs enables the identification of factors limiting the process’s effectiveness and the implementation of appropriate improvements in technological parameters [56,57,58]. These activities are essential when scaling the technology for industrial applications where high precision and repeatability are crucial. In this way, NIL technology can become not only a tool with significant scientific potential, but also a practical solution for the production of micro- and nanostructures with a wide range of industrial applications.

Nanoimprint lithography, being a high-precision, multi-step process in which the final results are heavily dependent on technological parameters, requires a shift from a traditional approach based solely on parameter control to one focused on results and process data. In this context, KPI modelling constitutes a fundamental element of the transformation in process management, allowing for a direct link between technological parameters and measurable outcomes in terms of quality, performance and stability.

The use of KPIs is also justified by the growing pressure to increase productivity, as well as the complexity and multi-variant nature of NIL technology. Appropriate indicators enable the implementation of multi-criteria optimisation, which combines quality and production objectives and ensures the comparability of different process variants. At the same time, they serve as a tool that integrates process data in line with the data-driven manufacturing approach, reduces quality costs, supports statistical process control and the implementation of Industry 4.0 solutions. As a result, KPIs form the foundation for the optimisation, effective control and further development of NIL technology in industrial applications.

For the effective implementation and scaling of NIL technology, it is reasonable not only to use KPIs as a control tool, but also to consciously model and adapt them to the critical stages and conditions of NIL processes and their individual variants, such as Thermal NIL, UV-NIL, Roll-to-Roll NIL, and Step-and-Repeat NIL. The development of adequate indicators requires consideration of both technological parameters and organisational factors that take into account the efficient use of resources. Depending on the variant of NIL technology, the emphasis should be placed on different groups of indicators. For example, in UV-NIL, parameters related to polymerisation kinetics and irradiation uniformity are of key importance, while in Roll-to-Roll processes, indicators related to process continuity, stress stability and repeatability of reproduction over large areas are important.

A properly designed KPI system can be a significant component of an integrated quality management model [59]. It will enable not only ongoing monitoring of process stability but also forecasting potential deviations and supporting decisions on production scaling [60,61,62]. The integration of an engineering approach with a management approach allows KPIs to be treated as a strategic tool that will support the long-term optimisation and improvement of NIL technology, which will facilitate its adaptation to diverse industrial requirements. For this reason, the further development of NIL technology should proceed in two directions: by improving material and process issues and, in parallel, by developing methods for conscious process management based on measurable and verified KPIs.

The KPI-based approach should be complemented by systematic quality control [63], which, within the framework of NIL technology, should cover both the mould preparation stage, the pattern mapping process, and the evaluation of the finished structure [55,64,65,66]. In this respect, KPIs are a tool for integrating measurement data with technological decisions. Systematic analysis of indicators will enable early detection of adverse trends, implementation of corrective measures and maintenance of the appropriate quality of replicated nanostructures [67,68,69]. Thus, the quality control process supported by an adequate set of KPIs becomes a significant element in ensuring the reliability, repeatability and, ultimately, competitiveness of NIL technology in industrial applications.

Currently, research on NIL technology and its variants focuses mainly on improving processes to achieve high resolution of nanoscale patterns, optimising material and process parameters, and increasing production efficiency [32,33,34,35,36,37,38]. This research analyses both macroscale continuum models for T-NIL and UV-NIL, as well as atomistic molecular dynamics simulations, which provide a better understanding of the impact of resist properties on imaging quality and defect minimisation [34,35,36,37,38]. Significantly less attention is paid to issues related to the conscious management of NIL processes, the only manifestation of which are research braces related to quality control [55,64,65,66]. Table 1 presents academic studies on the use of indicator-based analysis in fields related to NIL.

An analysis of the studies listed in Table 1 indicates that KPIs are applied across several complementary areas of nanotechnology and advanced manufacturing. They are used, among other things, in metrology and the evaluation of measuring equipment, where they serve to analyse the accuracy, sensitivity, and reliability of measurement systems [71], as well as in the evaluation of the performance of nanofabrication technologies and processes, covering aspects such as product quality, resolution, and scalability [72]. KPIs also play a significant role in research infrastructure management, enabling the comparison of operational models and the identification of key performance parameters [70], as well as in advanced, digitally controlled manufacturing (Industry 4.0), where they support process monitoring, quality control, and the optimisation of energy and cost efficiency [73].

At the same time, it should be emphasised that despite the widespread use of indicator-based analyses in related fields, the available literature lacks studies that directly and comprehensively address the application of KPIs in the context of nanoimprint lithography technology. This constitutes a research gap, especially in the context of technology scaling and its implementation in a mass production environment.

Filling the research gap is important not only for increasing process stability and repeatability, but also for ensuring the economic viability of NIL implementation. The creation of consistent process management models based on precisely defined KPIs can contribute to reducing technological risk, shortening implementation time, and improving process efficiency and resource utilisation. The integration of engineering tools with process management methods sets the strategic direction for further research, enabling the full exploitation of the potential of NIL technology and its effective adaptation in the modern micro- and nanostructure industry.

The objective of this study was to develop and model key performance indicators (KPIs) that support the comprehensive optimisation of nanoimprint lithography (NIL) processes, covering both the quality of nanoscale pattern reproduction, the stability of technological parameters, and production efficiency. This task involved identifying indicators relevant to technological practice and linking them to specific stages of the NIL process. In addition, the aim was to create an integrated KPI model enabling operational control of the process, assessment of the production capacity of various NIL variants (including Thermal NIL, UV-NIL, Roll-to-Roll NIL and Step-and-Repeat NIL) and supporting the implementation of a multi-criteria optimisation approach and statistical process control in industrial conditions. KPI modelling in this study provides in-depth knowledge on the optimisation and control of NIL processes, while offering useful guidance for scientists and engineers involved in the development of this technology.

The novelty of this study lies in shifting the focus of research on NIL technology from the previously dominant strictly technological perspective to an integrated engineering and management approach based on systematic modelling of an adequate set of KPIs. Unlike previous works, which focused mainly on optimising individual process parameters or selected aspects of reproduction quality, the proposed approach is comprehensive in nature. It includes a multidimensional KPI model linked to the individual stages of the NIL process, accounting for its variants (Thermal NIL, UV-NIL, Roll-to-Roll NIL, Step-and-Repeat NIL). The novelty of the study lies in the integration of quality, technological and production indicators into a single coherent operational model. The presented model enables simultaneous control of the quality of nanoscale structures, the stability of process parameters and production efficiency. In addition, the introduction of a multi-criteria approach and process control elements in the context of NIL process management represents a significant extension of the existing research paradigm. It creates a solid foundation for the conscious scaling of the technology and its effective implementation, followed by its implementation in an industrial environment.

## 2. Materials and Methods

Ensuring the reliability and effectiveness of measuring the efficiency of technological processes involves adopting an approach based on transparency, repeatability and wide availability of the analytical methods and tools used. The measurement system should be as general as possible in its scope of application to enable comparability of results in different organisational conditions and technological variants. It is important that the indicators created and the procedures for determining them are based on clearly defined criteria that are understandable to both a narrow group of experts and a wider group of stakeholders in the technological process.

An effective assessment system should also be based on solutions using components that are as open as possible, particularly with regard to analytical methods and the way results are reported. This means using unambiguous definitions of indicators, measurable parameters and standard data processing procedures, which helps to reduce interpretative subjectivity. The approach presented ensures transparency of the decision-making process and enables effective communication of results between technological, quality and management departments.

In the context of nanoimprint lithography processes, the ability of the measurement system to provide information on reproduction quality, technological parameter stability and operational efficiency is of key importance. The indicators should not only reflect the current state of the process, but also enable the identification of relationships between technological parameters and quality effects, supporting knowledge transfer and process improvement. In this regard, key performance indicators (KPIs) are a practical translation of quality and performance objectives into clearly defined, measurable parameters, forming the basis for objective evaluation and systematic optimisation of the NIL process.

**Figure 1:**
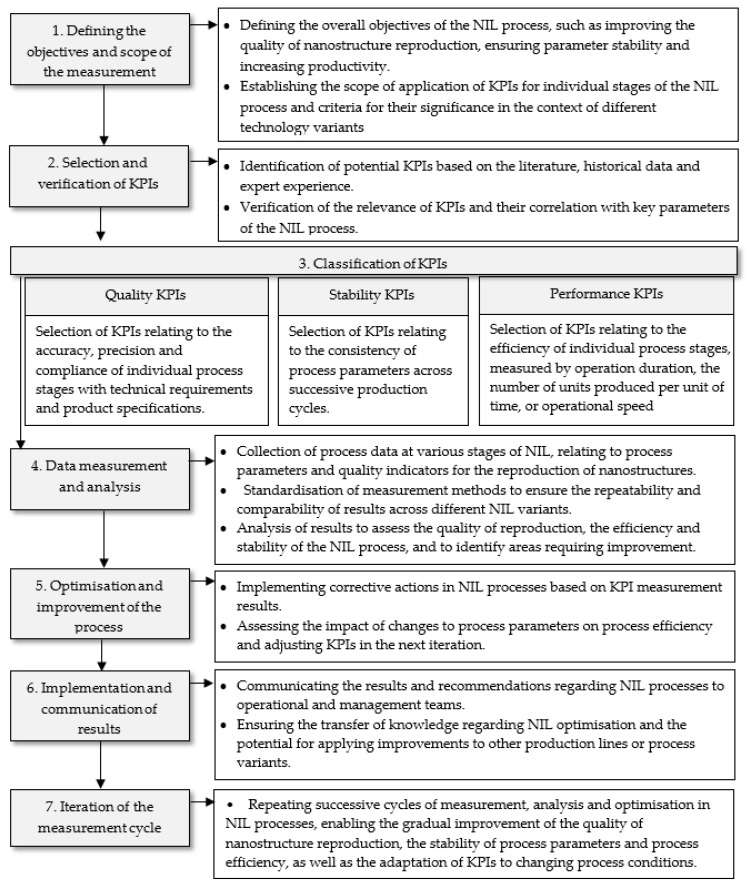
General iterative KPI modelling scheme supporting the optimisation of nanoimprint lithography (NIL) processes.

Based on the above premises and literature guidelines, an iterative model for measuring the effectiveness of NIL processes in industrial enterprises using KPIs was developed. The model assumes a continuous nature of measurement and improvement, combining the identification of KPIs, data collection, analysis of results and optimisation activities into a coherent performance management system. Figure 1 presents a general overview of the implementation of measurement processes according to the adopted model.

The procedure diagram shown in Figure 1 reflects seven repeatable stages enabling systematic monitoring and improvement of the quality of nanostructure mapping, stability of technological parameters and operational efficiency of the process. The first stage of the procedure involves defining process objectives and determining the scope of application of KPIs, including criteria for their relevance to different NIL variants (Thermal NIL, UV-NIL, Roll-to-Roll NIL, and Step-and-Repeat NIL). Next, in the stage of selecting and verifying indicators, potential KPIs are identified based on literature data, expert knowledge and experience, and previous process results. This step also involves verifying operational usability in the context of practical technological limitations. The third stage involves dividing the indicators into three groups: quality KPIs, stability KPIs and performance KPIs, and describing them. The fourth stage concerns the measurement and analysis of data. At this stage, data on quality and technological parameters is collected, and statistical data analysis methods are used to identify trends, deviations and potential sources of quality problems. The next stage involves optimisation activities consisting of implementing process corrections based on KPI results and assessing the impact of these changes on process stability and efficiency. The sixth stage concerns the implications of recommendations (based on the results obtained) in the area of production and operational practice, as well as ensuring knowledge transfer between technological, quality and management teams. This activity promotes the scalability of improvements. The entire cycle is closed by stage seven (measurement cycle iteration), in which successive rounds of measurement, analysis and optimisation support the continuous improvement of NIL processes and, at the same time, support the adaptation of KPIs to a changing technological and organisational environment.

The diagram illustrates a cyclical approach, within which data collection, analysis and corrective action are performed repeatedly, enabling continuous process improvement. The model emphasises the role of KPIs as tools not only for measurement but also for decision-making, allowing for a quantitative assessment of the impact of specific technological parameters on the final results and supporting the implementation of informed and appropriate optimisation measures.

An integral part of the model is also the transfer of knowledge and ensuring clarity of information. KPIs and their visual presentation enable the communication of results between technology, quality and management teams. This contributes to increased repeatability and scalability of the improvements introduced. In addition, the iterative nature of the model allows for the effective application of indicators to changing technological conditions, various NIL process variants and the specific characteristics of the production plant. The circular nature of the model provides a basis for integrating KPIs with statistical process control methods and multi-criteria optimisation. As a result, the presented model of conduct is not only a tool for monitoring effectiveness, but also a methodological framework for the systematic development of NIL processes in industrial and laboratory conditions.

The correctness and usefulness of the developed model were tested in one company located in Poland and operating in the modern electronics manufacturing sector. The company has been in operation for 22 years and employs just over 500 staff. It specialises in the manufacture of advanced electronic components, which are produced in mass- and medium-volume production runs using modern production lines characterised by a high degree of automation. Quality control, cost optimisation and operational efficiency play a key role in the production processes. A key element of the company’s operations is its advanced IT infrastructure, which enables the collection and analysis of production data in near real time. The company serves both the domestic market and international customers, which necessitates meeting high quality standards and, not infrequently, adapting flexibly to changing customer requirements. The specific nature of the industry, characterised by complex production processes and an extensive organisational structure, created the right conditions for verifying the developed model in economic practice. The analyses were carried out in the second and third quarters of 2025.

The company uses nanoimprint lithography technology in the UV-NIL variant, which was the direct reference point for the analyses performed. The use of this type of technology results from its ability to be integrated with existing production lines, its favourable operating parameters (especially high reproduction accuracy) and its energy efficiency.

To select an appropriate set of KPIs, a literature review was conducted, which identified the method developed by [74] that accounts for technical criteria and employee opinions. As part of the adopted methodology, questionnaires were used and interviews were conducted with employees performing key functions in the company. This allowed for the collection of information and opinions from people who have a direct impact on decision-making and process management. Survey questionnaires were distributed among department managers and some of the management staff, while interviews were conducted with employees in decision-making positions, responsible for formulating the company’s strategy and supervising its long-term development. This approach provides a practical and accessible method for identifying indicators by combining literature analysis with employees’ experience and expertise.

The survey questionnaire used in the study consisted of four thematically separate sections:

- The first part of the questionnaire contained the purpose of the study and detailed instructions on how to complete the survey. It explained how to answer the questions and the rating scale from 1 to 5, where 1 indicated very low importance of the indicator and 5 indicated very high importance. Its purpose was to provide respondents with clear instructions and a better understanding of the study.
- The second part of the survey included the respondent’s details, covering key demographic and professional information, i.e., position, department, length of service, level of education and age. The information collected enabled an analysis of the results, taking into account the differences among respondents.
- The third part of the questionnaire focused on the assessment of key performance indicators (KPIs) in the context of the individual stages of NIL process implementation.
- The fourth part of the survey covered the application of KPIs in different technology variants.

Both the third and fourth sections of the questionnaire identified specific stages of the NIL process and assigned them appropriate quality and performance indicators, enabling a quantitative assessment of the effectiveness and stability of the process. Each of the proposed KPIs was described in detail. The name of the KPI, its definition, calculation formula and a brief description indicating the essence of the indicator were included. This presentation enabled respondents to make an informed, comparable assessment of individual KPIs in terms of their usefulness and importance for maintaining an appropriate level of process quality and performance.

The questionnaire ended with open-ended questions, which gave respondents the opportunity to present their opinions, suggest additional indicators, and submit comments on the subject under study.

After completing the survey, the first step in selecting appropriate KPIs was to analyse the quantitative ratings given by respondents. For each indicator, the average rating and standard deviation were calculated to determine the level of agreement among the surveyed employees. KPIs with high average ratings and low levels of rating variance were considered particularly relevant and representative of the entire process.

The next stage involved analysing the results in the context of differences between groups of respondents, such as department, position or length of service. This made it possible to identify indicators that were considered key by all groups or by key production departments. Individual KPIs that were rated highly only in narrow groups were considered less representative and could be eliminated from the list of priority KPIs.

At the same time, information obtained from open-ended survey questions was also taken into account. Analysis of the responses enabled identification of additional indicators proposed by employees (reflecting the specific nature of the technological environment) and highlighted comments on difficulties or limitations in using certain indicators.

Based on the combined quantitative and qualitative results, KPIs were prioritised. The indicators were divided into three groups: key, supporting and optional. Key KPIs were rated highest and were relevant to all stages of the process and NIL technology variants. Supporting indicators were of moderate importance and were useful in specific stages or technology variants, while optional KPIs were of low importance or limited usefulness.

A total of 64 employees took part in the survey, of which 61 correctly completed questionnaires were included in the analysis. Three questionnaires were excluded from the analysis due to formal deficiencies or incomplete answers. Nevertheless, the response rate obtained should be considered high, which is confirmed by the satisfactory response rate (95.3%). The high response rate positively impacted the representativeness of the sample and increased the reliability of the results. The size of the research group was the result of the adopted methodological assumption that the survey was addressed only to employees directly involved in the implementation and supervision of NIL processes, as well as in the analysis and reporting of performance indicators.

The respondents mainly represented engineering and technological staff (41%) and persons performing managerial and supervisory functions (59%), responsible for the quality, stability and efficiency of nanoimprint lithography processes. The participants who took part in the survey were directly involved in the planning, implementation and control of individual stages of the process. Most of the respondents (68%) had a technical higher education, 21% had a technical secondary education, and 11% had other education. The average length of service at the company was 7 years, while the average experience in NIL technology and KPI monitoring was 3 years.

The study participants represented various functional areas related to the implementation of NIL processes, including: substrate and resist layer preparation, imprinting (Thermal and UV), curing and matrix separation processes, quality control of nanometric structures, and support departments such as maintenance, environmental parameter control, production planning and process data analysis. This functional diversity of respondents provided a comprehensive picture of the importance of individual KPIs throughout the entire technology chain.

To supplement the survey results of the survey and deepen the analysis of NIL processes and the applicability of individual KPIs, qualitative analysis was conducted through in-depth interviews. In this case, too, the selection of respondents was purposeful and included representatives of engineering and management staff directly involved in the implementation, supervision and optimisation of individual stages of the NIL process. The interviews were conducted in a semi-structured format, referring to the thematic division of the survey questionnaire, which ensured the comparability of the scope of the interviews while allowing for the exploration of issues that were important from the perspective of operational practice.

The scope of the in-depth interviews included the identification of key factors determining the stability and effectiveness of the process, the assessment of the adequacy and usefulness of the analysed KPIs in production conditions, and the identification of potential limitations related to their implementation and monitoring. The collected information (qualitative material) was subjected to content analysis, which allowed for an in-depth interpretation of the survey results, verification of the accuracy of the selected indicators, and clarification of their significance in various variants of NIL technology. The integration of quantitative and qualitative research results contributed to a more comprehensive and methodologically consistent picture of the issue under study.

The final selection of KPIs took into account survey statistical results, practical comments from respondents, and information gathered from in-depth interviews. As a result, the selected KPIs were relevant to the efficiency and quality of NIL processes, and at the same time were measurable, comparable and consistent with the experience and expectations of the operational team. This approach ensured a reliable and objective determination of the most important KPIs for the processes under study.

The general nature of the model means that the results achieved are not limited to the analysed cases of industrial enterprises. This makes it possible to apply the developed relationships and recommendations to other organisations with similar operating characteristics, process structures or technological conditions, which contributes to increasing the application value of the developed model.

The presented approach to modelling key performance indicators (KPIs) in the context of optimising nanoimprint lithography (NIL) processes was the starting point for the next stage of the analysis. It enabled the presentation of the obtained results and their in-depth interpretation from the perspective of rational and conscious management of production processes.

## 3. Results and Analysis

### 3.1. Division of Nanoimprint Lithography Processes

This section presents the division of nanoimprint lithography processes in industry. Taking into account the stages of the NIL process and its various technological variants is the starting point for the taxonomy of KPIs for individual activities undertaken in the field of improvement and ensuring an appropriate level of quality. This organisation of processes allows for the unambiguous assignment of indicators to specific technological stages, which increases the transparency of the performance measurement system. It also allows for the identification of differences in the significance of individual KPIs across the technology variants used. As a result, it supports a more precise matching of monitoring tools to the specifics of the process. Consequently, the adopted classification forms the basis for a coherent, hierarchical system for assessing quality and performance in the NIL environment.

Before proceeding with the analysis of KPIs and the assessment of NIL process efficiency, it is important to present the structure of the technological process. To this end, NIL processes have been organised into stages, which allows them to be compared in the context of different technology variants and to identify critical points for quality and performance. Table 2 presents the general stages of the nanoimprint lithography process in terms of technological variants: Thermal NIL, UV-NIL, Roll-to-Roll NIL and Step-and-Repeat NIL.

Table 2 summarises of the stages of the NIL process across different technological variants. It should be noted that the summary includes a generalised list of key stages of the process that are most important for ensuring the quality and efficiency of the technology, rather than all detailed technological operations.

The ‘further technological processing’ stage may include activities preparing the substrate for subsequent product processing, such as etching, metallisation, thin film deposition or integration with other components in the final product. These activities no longer create the nanostructure itself, but ensure that the product is ready for further use and enable quality control of the nanostructures in the context of the entire product. For this reason, this generalised stage of the process concludes the characteristics in Table 2.

Certain specific actions and activities for individual NIL process variants, such as minor preparatory activities, equipment adjustments, detailed parameters for resist layer application, or additional integration operations after imprinting, have not been included. This simplification has been deliberately applied to ensure a transparent comparison of technological variants across the main stages of the process. The omitted operations are usually carried out as part of standard operating procedures within the company and do not change the overall course of the NIL process. It is not reasonable to include them in separate indicators. Minor operational activities do not usually provide information that is important for the quality and efficiency of the entire process. An excess of KPIs can lead to distraction and make it difficult to identify the causes of deviations.

An extensive KPI system, which also takes into account activities with a marginal impact on the final result, expands the structure of the reporting system, generates additional costs of data collection, monitoring and analysis, and may also result in excessive information overload. As a result, it hinders effective management by indicators, which is essentially focused on key, measurable elements that determine the stability of the process. For this reason, the analysis takes a selective approach, focusing on stages that have a real impact on the quality of structure reproduction and the effectiveness of NIL technology.

As part of the systematisation of the analysis of processes performed in nanoimprint lithography technology, they were divided into three main groups: main processes, auxiliary processes and management processes. The classification makes it possible to distinguish between activities directly related to the shaping of the product structure, activities supporting the course of technological operations, and activities of an organisational and decision-making nature. The adopted division is presented in Table 3 in relation to individual NIL technology variants. This allows for a clear presentation of the process structure and identification of differences and similarities between technological solutions.

The ‘further technological processing’ stage refers to activities preparing the substrate for further operations on the product, in which the nanostructure has already been created in the NIL process. In practice, this means that these activities concern subsequent stages of product manufacturing, rather than further stages of the NIL technology itself, and thus may serve as auxiliary or management processes. Considering this fact, both functions were analysed in relation to the ‘further technological processing’ process, thus creating two sets of KPIs—the first in terms of the auxiliary process, the second in terms of the management process.

Table 3 provides a clear overview of the stages of the NIL process, taking into account the variety of technological variants and the importance of individual activities for the quality, stability and efficiency of the process in terms of the process itself. At the same time, it provides a starting point for KPI taxonomy, allowing for the design of adequate indicators to improve processes and ensure an appropriate level of quality, stability and efficiency.

### 3.2. KPI Taxonomy in Relation to Nanoimprint Lithography Processes

As part of the study, the scope of KPI modelling was also extended to other variants of NIL technology, namely Thermal NIL, Roll-to-Roll NIL and Step-and-Repeat NIL. This approach is general in nature and allows for the development of a universal KPI taxonomy structure that can be applied in organisations using different technological solutions. The expansion of the scope of analysis also allows for the identification of common and specific critical areas in terms of quality, stability and process efficiency, depending on the technological variant. This increases the practical usefulness and implementation potential of the developed model.

The objectives for the analysed NIL technology variants concern increasing the efficiency of the processes implemented, which consists of ensuring the quality, stability and performance of the processes. This is characterised in relation to the KPIs in Table 4.

An integrated approach to KPIs in NIL technology, involving simultaneous monitoring of three groups of indicators, namely quality, stability and performance KPIs, supports comprehensive and consistent process management. This approach supports the achievement of process objectives by identifying critical stages in terms of product quality, locating sources of process stability fluctuations and implementing measures to improve operational efficiency. At the same time, it reduces the risk of an undesirable decline in end parameters, ensuring a balance between high quality, repeatability and productivity of the NIL process.

Based on the adopted taxonomy of NIL process stages, survey results and in-depth interviews, a preliminary set of potential key performance indicators (KPIs) was developed, relating to process quality, stability and efficiency. The proposed indicators were then verified in consultation with representatives of the management of a company using UV-NIL technology. Table 5 presents a set of KPIs for auxiliary processes in the context of ensuring the quality, stability and efficiency of processes, taking into account all variants of NIL technology.

Table 5 describes the KPIs for the ‘substrate preparation’ stage. The proposed KPIs focus on ensuring appropriate input conditions for further NIL operations. Quality indicators relate to cleanliness and defects, stability indicators relate to the repeatability of process parameters, and performance KPIs enable the assessment of operational efficiency. Together, they help reduce defect risk in subsequent stages. In contrast, for the ‘further technological processing’ stage (in terms of the auxiliary process), the KPIs focus on maintaining the final parameters and pattern transfer fidelity. Stability indicators allow for the control of process variability, while performance indicators allow for the control of operational efficiency. This system supports quality maintenance and optimisation of the final phase of the NIL process.

The review of the literature, surveys and direct interviews also resulted in the development of KPIs for the main NIL processes. The characteristics of the proposed KPIs are presented in Table 6.

Table 6 presents a comprehensive overview of KPIs for the main processes within the NIL process. The structure of the indicator set correlates with the individual stages of the processes. The developed list of KPIs accounts for differences in the implementation of NIL processes in individual technological variants, identifying indicators related to key differences and critical activities. This allows us to track how the quality of nanostructure mapping is shaped at subsequent stages of implementation. At the same time, it enables control of the stability of technological parameters and assessment of the effectiveness of individual operational activities. This makes it possible to examine the impact of individual stages on the outcome of the process and to assess the repeatability of the process under different technological conditions. This system allows us to examine the links between individual aspects of the process evaluation. It also allows us to identify the stages that are most important for efficiency (quality, stability, and performance) and to identify potential areas for optimisation or improvement.

Due to the specific nature of the “further technological processing” stage, it has been divided into the implementation of the process in auxiliary and managerial contexts. This division into auxiliary and managerial approaches is justified by the multidimensional nature of the NIL process. This stage includes both operational technological activities that directly affect the product’s structure and supervisory and integrative functions within the production system. In terms of support, KPIs focus on the quality, stability, and efficiency of technological operations, while in terms of management, they focus on the coordination of activities, the implementation of plans, and the efficient use of resources. This separation enables us to model process efficiency at both the technological and organisational levels, creating a coherent set of indicators to support the improvement of the NIL process. Therefore, a set of KPIs has also been developed for this process from a management perspective (Table 7).

The summary in Table 7 synthesises KPIs for the further technological processing stage, organising them into three complementary dimensions: quality, stability, and efficiency. Quality indicators focus on the degree of goal achievement and the level of non-compliance, providing a basis for assessing the effectiveness of process supervision. Stability indicators relate to the repeatability of schedule implementation and the consistency of audit results, which allows for the verification of organisational maturity and control mechanisms at a given stage of the process. In turn, performance KPIs integrate the assessment of resource utilisation (OEE), production plan implementation, and response speed to non-compliance, integrating the operational and management perspectives. As a result, this structure is a tool that supports systematic monitoring of the process and the development of optimisation measures at the management level.

The standardisation of units across separate KPI groups forms the basis for building a consistent system for evaluating NIL processes across variants. With regard to qualitative KPIs (Table 5, Table 6 and Table 7), it was decided to express them as a percentage of compliance with the specification or as the number (density) of defects per batch or unit area. The approach used allows for: direct comparison of individual stages and variants of NIL, standardisation of results regardless of production volume, and aggregation of indicators in models evaluating the process as a whole. At the same time, it reflects the regulatory dimension (compliance with specifications) and the technological dimension (actual level of non-compliance).

In terms of KPIs for stability (Table 5, Table 6 and Table 7), standardisation to a percentage of variability was adopted. This approach eliminates the problem of different physical units, allows for the comparison of the stability of different parameters on a single scale, and provides a basis for developing synthetic measures of stability. Stability was treated as a measure of the process’s ability to maintain parameters within tolerance limits.

With regard to performance indicators (Table 5, Table 6 and Table 7), unit time (e.g., minute/unit) or productivity per unit of time/area (e.g., unit/hour, square metre/hour) was used. This made it possible to directly assess the productivity of a process stage, compare operating modes (continuous versus batch), and integrate KPIs with cost analysis and OEE (Overall Equipment Effectiveness) indicators. The assumptions and division adopted ensure the analytical consistency of the system and its usefulness in operational and strategic terms.

The broad and extensive approach to KPIs (Table 4, Table 5 and Table 6) results from the fact that the nanoimprint lithography technology used in the UV-NIL variant is new and qualitatively unstable in the company, which requires detailed monitoring of all critical activities and parameters. As the quality and repeatability of the process stabilises, it will be possible to gradually reduce the number of KPIs to the most relevant ones, which will simplify monitoring and reporting. It is worth noting that the current approach, based on a relatively large set of indicators, also ensures greater versatility of the KPI model and allows for its potential adaptation in other companies.

The study takes into account both indicators that can be measured and calculated relatively easily within the production environment, and those that are more difficult to observe or calculate directly. The first group primarily comprises operational indicators, relating, for example, to the duration of key activities within the process, productivity, or the repeatability of technological parameters. Their ongoing monitoring enables rapid detection of deviations and allows for immediate corrective action, making them particularly useful in production control and ensuring process stability.

At the same time, qualitative KPIs have been included, measured through indirect or periodic analyses or the use of statistical process control methods, such as defect density or the degree of nanostructure reproduction. These indicators serve a diagnostic and optimisation function, supporting process validation, analysis and the planning of improvement measures. This hybrid approach enables the integration of real-time measurable data with the results of off-line analyses. This is consistent with the concept of data-driven manufacturing. At the same time, it enables the identification of a subset of KPIs with high measurability, which can be applied to practical industrial implementations.

The optimisation and improvement of the NIL process should be based on systematic KPI analysis. Based on the results, the causes of deviations are identified and precise corrective actions are implemented, which may involve modifying the temperature, pressure, exposure time, or substrate preparation, while maintaining control over individual factors. The effectiveness of the changes is assessed by re-measuring the parameters and comparing the results with previous cycles.

The implementation and communication of results in NIL processes should be orderly and systematic. The results achieved (KPI values and conclusions drawn from them) and recommendations for process improvement should be communicated to both the operational teams responsible for day-to-day production control and the management staff responsible for planning and strategic decisions. Communication should ensure a clear presentation of the impact of the implemented changes on the quality, stability, and efficiency of nanostructure mapping. It is also important to ensure knowledge transfer within the organisation. Knowledge transfer should occur between different production lines and between variants of NIL technology, thereby contributing to the effective implementation and replication of proven improvements in other processes, increasing the efficiency and repeatability of the entire production system.

The iteration of the measurement cycle within the framework of NIL process management framework using KPIs should be carried out systematically and continuously. Each measurement cycle involves collecting current and relevant process data, analysing it to assess the quality of nanostructure mapping, the stability of technological parameters, and process efficiency, and if necessary, introducing appropriate corrective measures. The results of the measures taken should be evaluated, and the indicators should be adjusted to new technological conditions. Each subsequent iteration enables the gradual improvement of critical stages of the process, as well as increased repeatability and precision of nanoscale pattern mapping, and ensures that individual KPIs remain relevant and useful in a changing production environment.

As part of the study, the scope of KPI modelling was also extended to other variants of NIL technology, namely Thermal NIL, Roll-to-Roll NIL, and Step-and-Repeat NIL, for which KPIs were modelled with the same level of detail. This expansion is justified from a methodological point of view, as all NIL technologies show significant similarities in terms of process structure, critical stages, and main operations, which allows for the identification of identical stages that are crucial for efficiency in terms of quality, stability, and process performance. Taking technological differences into account makes it possible to identify specific activities that are critical for each variant, which increases the practical usefulness of the analysis. The approach used is general in nature and allows for the creation of a universal KPI taxonomy structure that can be applied in organisations and institutes using different technological solutions.

## 4. Discussion

Nanoimprint lithography (NIL) is a modern technology for producing nanostructures. It involves mechanically reproducing the mould’s topography in a resist layer. It is considered a promising alternative and complement to conventional lithographic techniques, as it enables the production of very high-resolution patterns at relatively lower infrastructure costs [18,21,22].

The literature on NIL technology indicates that research focuses largely on improving its material and process aspects across variants, such as T-NIL and UV-NIL [32,33,34,35,36,37,38]. This includes both experimental optimisation of technological parameters (such as temperature, pressure, process time) [32] and numerical modelling using continuum mechanics [33,36,37]. Molecular dynamics simulations are conducted to analyse phenomena occurring at the atomic scale, which are particularly important at resolutions below 10 nm [34,35,38]. Another significant area of research is the assessment of the impact of the physicochemical properties of resists (such as viscosity, polymer chain length, and molecular composition) on the quality of mould cavity filling and the presence of incompatibilities [34,35,36,37,38]. The aim of the work is to increase imaging resolution, improve process stability, and expand the applications of NIL technology in the production of photonic and electronic products [41,42,43,44].

An important, albeit insufficiently developed area of research and analysis is the quality control of NIL processes, including the assessment of pattern accuracy, analysis of non-compliance, and stability of technological parameters within individual stages of the process [55,64,65,66]. The literature points to the need for systematic quality monitoring and integration of measurement data with technological decisions [63,67,68,69]. Key performance indicators (KPIs) are used in fields related to nanoimprint lithography, including metrology and the evaluation of measuring equipment [71], the assessment of nanofabrication process efficiency and product quality [72], research infrastructure management [70], and digitally controlled manufacturing in industry 4.0 [73]. At the same time, there is a noticeable research gap regarding a comprehensive approach to NIL process management using KPIs. Previous research has focused mainly on technological issues. There is a lack of studies that present the integration of the engineering perspective with the management approach, which would enable the modelling of process-efficiency assessment systems, especially in the context of their scaling and implementation in production space.

The implementation of appropriately selected KPIs for each stage of the NIL process enables systematic and precise monitoring of the entire manufacturing cycle. This approach allows for the identification of stages that are critical to quality, stability, and process efficiency, and also supports the assessment of savings potential and decision-making on improvement and technological modernization in areas where they can deliver measurable benefits. Thanks to the information provided by comprehensively selected KPIs, it is possible to compare parameters both across different production lines and within successive production shifts, thereby increasing the usefulness of the tool in process management. Among the additional advantages of the presented procedure within the framework of KPI modelling in NIL process optimisation, the following long-term benefits can be distinguished:

- Support in the systematic improvement of NIL processes by identifying patterns in data on quality, stability, and performance for individual technology variants;
- Support in planning technological upgrades in areas where changes in process parameters will have the greatest effect in terms of performance and mapping quality;
- Support in the development of predictive models that forecast the effects of parameter changes on specific NIL variants, supporting strategic decisions on the selection of technologies for specific applications and production requirements;
- Creation of a basis for standardising the assessment and monitoring of KPIs throughout the enterprise, taking into account the specificity of NIL variants, which improves the organisational and control capacity of processes.

In the short term, the implication of KPI modelling in NIL processes brings the following benefits:

- Precise monitoring of parameters specific to individual NIL variants;
- Rapid identification of critical stages in terms of nanostructure reproduction quality and process stability, which is important in processes that require high repeatability of micro- and nanostructure reproduction;
- Comparison of process performance and stability between individual NIL technology variants and production lines, which supports the selection of the most effective technologies;
- Ongoing support for decisions regarding the optimisation of technological parameters specific to individual NIL variants, minimising the risk of deviations from the assumed standards.

The presented approach to KPI modelling, linked to their taxonomy, provides ongoing operational support. At the same time, it brings strategic benefits in terms of improving the NIL process, taking into account the diversity of technological variants and their impact on the quality, stability, and efficiency of production. However, the presented procedure is associated with certain limitations, which include:

- Limited standardisation between companies—not all organisations have the same set of input data needed to calculate KPIs;
- The number of indicators depends on the stability of the process—a high degree of KPI granularity is justified in the case of processes that are unstable in terms of quality (such as UV-NIL in the company in question). Once the process has stabilised, it becomes appropriate to reduce the number of KPIs used within the process, retaining only those necessary for monitoring the process;
- Subjectivity of the assessment of some of the proposed qualitative KPIs—measuring the quality of nanostructures (e.g., pattern integrity, defect density) may require expert interpretation or the use of visual/inspection methods, which introduces an element of subjectivity.

A limitation of the presented procedure for KPI modelling as part of NIL process optimisation is the fact that a lack of adequate staff training and possible organisational resistance may result in incorrect implementation and application of the developed model.

The procedure presented in the article for modelling key performance indicators as part of NIL process optimisation makes a significant contribution to the systematic optimisation of technology. It enables both the identification of critical process stages and the assessment of the relationship between the quality of nanostructure mapping, the stability of process parameters, and the level of process efficiency. The developed model allows for precise monitoring and comparison of different NIL technology variants, as well as for the design of dedicated improvement measures. In practice, the study can be used by research laboratory managers, quality and production specialists, process engineers, and managers responsible for the implementation of advanced lithography technologies. The presented procedure can support day-to-day operational decisions as well as strategic process development planning, while providing tools for effective monitoring, analysis, and optimisation.

Future research will focus on modelling risk and uncertainty in NIL processes using KPIs. The research will include developing predictive indicators for early identification of failure events and detecting fluctuations in machine and material parameters that may reduce the quality of nanostructures or destabilise the process. In particular, we plan to conduct an in-depth analysis of parameters such as: imprint pressure, process temperature, cycle time, material viscosity, and polymer deformation rate, as well as the roughness and surface properties of the mould. In addition, parameters relating to pattern reproduction accuracy, structural uniformity and defect rates will be taken into account. Optimisation of the specified parameters will be carried out using statistical analysis methods, regression models and optimisation algorithms, which will enable the determination of their optimal ranges. Plans for future research also include the use of machine-learning-based approaches to identify non-linear relationships between process parameters and KPIs. The research will focus on analysing the relationship between deviations in process parameters and changes in the values relevant to individual KPIs, which will enable the creation of predictive models to support risk management and stabilise the technological process.

## 5. Conclusions

Nanoimprint lithography processes are characterised by high sensitivity to variations in technological parameters and a multi-stage structure, which requires systematic monitoring of their progress and effectiveness. For this reason, the aim of the study was to model key performance indicators to optimise the quality, stability, and efficiency of nanoimprint lithography processes.

The research showed that the developed model is logically sound. Verification of the model under industrial conditions confirmed its practical usefulness, enabling effective monitoring and optimisation of processes. The research confirmed that KPI modelling is a useful solution for optimising nanoimprint lithography processes under nanomanufacturing conditions. The integrated KPI model, directly linked to both process stages and the critical technological parameters of various NIL variants (Thermal, UV, Roll-to-Roll, Step-and-Repeat), enables simultaneous assessment of process quality, stability, and efficiency. The use of a process analysis approach, together with multi-criteria optimisation, has enabled the systematisation of key production efficiency indicators and their linkage to measurable quality effects. The implementation of an integrated KPI model increases process efficiency (throughput) while maintaining the required quality parameters and technological stability.

The presented solution makes a significant contribution to the development of mechanical engineering and nanomanufacturing technology by introducing of a universal KPI modelling structure adaptable to diverse production environments.

## Figures and Tables

**Table 1:** Research papers relating to the use of indicators in fields related to NIL.

Area	Characteristics	Autor
Management of research infrastructure in nanotechnology using indicators	An empirical review of research infrastructures involved in the production and characterisation of nanomaterials (NFCRI), focusing on an analysis of the similarities and differences in their operational models. On this basis, key operational parameters are identified which can serve as a foundation for formulating and evaluating key performance indicators (KPIs).	Anson et al., 2022 [70]
KPIs in metrology and the evaluation of measurement systems in nanotechnology	Presentation of an advanced system of non-invasive measurement equipment in nanotechnology, focusing on improving the quality, accuracy and reliability of scanning and data analysis. It highlights the importance of performance metrics, such as sensitivity, immunity to interference and error propagation, in the context of modern measurement systems and the IoT.	Azeemi, 2021 [71]
KPIs in the evaluation of the performance and quality of nanophotonic (plasmonic) technologies and their manufacturing processes	The text presents the development of plasmonic colours as an advanced nanophotonic technology, comparing it with traditional solutions using key performance indicators (KPIs). It emphasises the importance of parameters such as resolution, scalability and compatibility of manufacturing processes, which form the basis for assessing the effectiveness and application potential of this technology.	Kristensen et al., 2017 [72]
KPIs in the digitally controlled production of nanostructured functional surfaces (Industry 4.0)	This study presents a concept for the scalable fabrication of bio-inspired surfaces using various lithographic techniques, integrated within a data-driven approach. Standardised key performance indicators (KPIs) play a key role, enabling the linking of process parameters to product functionality and ensuring control, repeatability and energy efficiency in production.	Chae and Kang, 2026 [73]

**Table 2:** Stages of implementation of individual variants of nanoimprint lithography processes in general terms.

Process Stage	Thermal NIL	UV-NIL	Roll-to-Roll NIL	Step-and-Repeat NIL
substrate preparation	cleaning, adhesion modification, surface control	cleaning, adhesion modification, surface control	preparation of a flexible substrate in a roll	preparation of silicon or glass substrates in batches
resist coating	spin-coating or other method of thermoplastic application	application of liquid photopolymer	coating on a flexible substrate in a roll	spin-coating or nano-coating on a rigid substrate
contact and imprint	die pressure, heating above polymer (Tg)	matrix pressing, UV curing	continuous pressure of the matrix during roll movement, heating or UV	matrix pressing in batch mode, optional heating or UV
structure fixation	cooling to harden the polymer	UV polymerization	cooling or UV curing in a roll	cooling or UV curing in stages
matrix separation	die separation after cooling	matrix separation after UV curing	continuous separation of the matrix	separation of the matrix after each imprint step
residual layer removal	plasma or chemical etching	plasma or chemical etching	continuous etching in a roll	etching in stages in batches
further technological processing	proper etching, metallization, integration	etching, thin film deposition	continuation of processes in a roll, e.g., transfer to other substrates	etching, metallization, integration of nanostructure batches

**Table 3:** Structural classification of the stages of the nanoimprint lithography (NIL) process, taking into account the type of process and the function of each stage from the analysed technological variants.

Process Type	Stage of the Process	Key Remarks	The Objective of the Stage in Terms of Quality, Stability, and Process Efficiency
auxiliary process	preparation of the substrate	ensuring cleanliness and adequate adhesion of the substrate; in Roll-to-Roll, preparation of a flexible roll; in Step-and-Repeat, standardisation of substrate batches	surface stabilisation and minimization of defects in further stages of the process
main process	application of a resist layer	Thermal and Step-and-Repeat—spin-coating, UV—liquid photopolymer, Roll-to-Roll—coating in motion	obtaining an even layer of resist preparing the substrate for imprinting
	contact and imprint	Thermal—heating, UV—UV light polymerization; Roll-to-Roll—continuous imprint, Step-and-Repeat—step-by-step	accurate reproduction of the matrix pattern on the resist layer
	fixing of the structure	Thermal—cooling, UV—polymerization; Roll-to-Roll—curing in motion, Step-and-Repeat—step-by-step	ensuring the durability of nanostructures and process repeatability
	separation of the matrix	In Roll-to-Roll motion synchronisation, Step-and-Repeat—batch control; Thermal and UV—after curing	separation of the matrix with minimal risk of pattern damage
	removal of the residual layer	plasma or chemical etching; Roll-to-Roll—continuous process, Step-and-Repeat—step-by-step	elimination of the thin resist layer and preparation of the substrate for further processing
support process/management process	further technological processing	actual etching, metallization, thin film deposition, integration; differences in operating mode (continuous vs. step-by-step)	readiness of the substrate for subsequent processes and control of final parameters

**Table 4:** Characteristics of the correlation between the objectives adopted in NIL technology and key performance indicators.

Quality KPIs	Stability KPIs	Performance KPIs
The indicators refer to the accuracy, precision and compliance of individual stages of the process with technological requirements and product specifications. In the case of NIL, these include the fidelity of nanostructure reproduction, the uniformity of resist layer thickness and the absence of defects after matrix separation.	The indicators measure the repeatability of process parameters in successive production cycles. This applies to both main stages, such as imprinting or structure fixing, and auxiliary stages, e.g., substrate preparation.	Indicators assess the effectiveness of individual stages of the process, measured by the duration of the operation, the number of products per unit of time or the speed of operation (e.g., roll speed in Roll-to-Roll NIL).
Quality KPIs allow us to assess the extent to which the process meets the design assumptions and what the potential risks of defects are. Monitoring them is crucial to ensuring high-quality end products.	High process stability is essential to ensure repeatable quality and minimise defects when producing larger numbers of products.	Efficiency is particularly important in the context of optimising production costs and shortening the technological cycle, while maintaining quality requirements.

**Table 5:** List of auxiliary process indicators in the context of ensuring quality, stability and efficiency of processes.

Stage of the Process	NIL Variants	Quality KPIs	Stability KPIs	Performance KPIs
preparation of the substrate	all NIL variants	surface cleanliness compliance (%); defect density (%)	variability of adhesion parameters (%); variability of roughness (%)	preparation time (%); capacity (%)
further technological processing		compliance of final parameters with specifications (%); pattern transfer accuracy (%)	variability of process parameters (%); repeatability of results between batches (%)	stage completion time (%); operational capacity (%)

**Table 6:** List of key process indicators in the context of ensuring quality, stability, and efficiency of processes.

Stage of the Process	NIL Variants	Quality KPIs	Stability KPIs	Performance KPIs
Applying a layer of resist	Thermal, Step-and-Repeat	layer thickness conformity (%); coating defect density (%); application accuracy (%)	layer thickness variability (%); spin speed stability (%)	application time (%); productivity (%)
	UV	photopolymer thickness conformity (%); coating uniformity (%); application accuracy (%)	viscosity repeatability (%); UV power stability (%)	application time (%); productivity (%)
	Roll-to-Roll	coating uniformity (%); defect density (%); application accuracy (%)	coating stability (%); web tension variability (%)	line speed (%); surface productivity (%)
Contact and imprint	Thermal, Step-and-Repeat	pattern reproduction accuracy (%); imprint defect density (%)	pattern dimension variability (%); temperature and pressure stability (%)	cycle time (%); number of cycles (%)
	UV	pattern transfer accuracy (%); defect density (%)	polymerization stability (%); UV dose variation (%)	cycle time (%); throughput (%)
	Roll-to-Roll	pattern accuracy (%); linear defects (%)	pressure stability (%); dimension variation (%)	production speed (%); throughput (%)
Consolidation of structure	Thermal, Step-and-Repeat	degree of fixation (the material’s ability to retain its shape after the mould has been removed) (%); structural integrity (%)	temperature variation (%); process stability (%)	fixing time (%); output (%)
	UV	degree of curing (%); mechanical property compliance (%)	curing repeatability (%); exposure stability (%)	exposure time (%); output (%)
	Roll-to-Roll	curing uniformity (%); defect density (%)	process parameter stability (%); speed variation (%)	line speed (%); output (%)
Matrix separation	Thermal, UV, Step-and-Repeat	absence of defects after separation (%); geometry conformity (%)	peel strength stability (%); process repeatability (%); service life of the die (%)	separation time (%); productivity (%)
	Roll-to-Roll	pattern integrity (%); linear defects (%)	roll tension stability (%); parameter variability (%); service life of the die (mould/stamp) (%)	roll speed (%); productivity (%)
Removal of the residual layer	all NIL variants	removal efficiency (%); residual thickness conformity (%)	plasma parameter stability (%); process repeatability (%)	etching time (%); productivity (%)

**Table 7:** List of management process indicators in the context of ensuring quality, stability, and efficiency of processes.

Stage of the Process	NIL Variants	Quality KPIs	Stability KPIs	Performance KPIs
further technological processing	all NIL variants	compliance with the quality plan (%); internal complaint rate (%)	variability in schedule implementation (%); repeatability of audit results (%)	OEE stage (availability × performance × quality) (%); production plan fulfilment (%); response time to non-compliance (%)

## Data Availability

The original contributions presented in this study are included in the article. Further inquiries can be directed to the corresponding author.
